# Breaking the Hardness-Wear Trade-Off: Quantitative Correlation in Nano-Al_2_O_3_-Reinforced Al_10_Cr_17_Fe_20_NiV_4_ High-Entropy Alloys

**DOI:** 10.3390/nano15100775

**Published:** 2025-05-21

**Authors:** Cong Feng, Huan Wang, Yaping Wang

**Affiliations:** 1 MOE Key Laboratory for Nonequilibrium Synthesis and Modulation of Condensed Matter, School of Physics, Xi’an 710049, China; 2 Beijing Shenzhou Aerospace Software Technology Co., Ltd., Beijing 100094, China; wanghuan1217@bjsasc.com

**Keywords:** multi-principal element alloys, hardness, wear resistance, nano-Al_2_O_3_ reinforced

## Abstract

Multi-principal element alloys (MPEAs) exhibit distinct characteristics compared to conventional single-principal element-based metallic materials, primarily due to their unique design, resulting in intricate microstructural features. Currently, a comprehensive understanding of the fabrication processes, compositional design, and microstructural influence on the tribological and corrosion behavior of multi-component alloys remains limited. While the hardness of MPEAs generally correlates positively with wear resistance, with higher hardness typically associated with improved wear resistance and reduced wear rates, quantitative relationships between these properties are not well established. In this study, the Al_10_Cr_17_Fe_20_NiV_4_ alloy was selected as a model system. A homogeneous Al_10_Cr_17_Fe_20_NiV_4_ alloy was successfully synthesized via mechanical alloying followed by spark plasma sintering (SPS). To further investigate the correlation between hardness and wear rate, varying concentrations of alumina nanoparticles were incorporated into the alloy matrix as a reinforcing phase. The results revealed that the Al_10_Cr_17_Fe_20_NiV_4_ alloy exhibited a single-phase face-centered cubic (FCC) structure, which was maintained with the addition of alumina nanoparticles. The hardness of the Al_10_Cr_17_Fe_20_NiV_4_ alloy without nano-alumina was 727 HV, with a corresponding wear rate of 2.9 × 10^−4^ mm^3^·N^−1^·m^−1^. The incorporation of nano-alumina increased the hardness to 823 HV, and significantly reduced the wear rate to 1.6 × 10^−4^ mm^3^·N^−1^·m^−1^, representing a 45% reduction. The Al_2_O_3_ nanoparticles effectively mitigated alloy wear through crack passivation and matrix strengthening; however, excessive addition reversed this effect due to the agglomeration-induced brittleness and thermal mismatch. The quantitative relationship between hardness (HV) and wear rate (W) was determined as W = 2348 e^(−0.006HV)^. Such carefully bounded empirical relationships, as demonstrated in studies of cold-formed materials and dental enamel, remain valuable tools in applied research when accompanied by explicit scope limitations.

## 1. Introduction

The development of novel, environmentally benign metallic materials is paramount for achieving sustainable development goals by minimizing resource consumption and mitigating environmental pollution. As the foundational components of modern society, metallic materials are indispensable for technological advancement and the enhancement of living standards, driven by continuous innovation [1,2,3,4,5]. The increasing diversification of application demands necessitates the exploration of new alloy concepts to overcome the limitations of existing alloy systems and foster advancements in materials science [6,7,8,9,10,11]. Pioneering work by Yeh Junwei [12] and Cantor [13] in Taiwan introduced the concept of multi-principal element alloys (MPEAs), which revolutionized the alloy design. MPEAs typically comprise five or more principal elements, each present in concentrations of approximately 5–35 at%, synergistically influencing the alloy’s structure and properties. These alloys exhibit unique characteristics not observed in conventional alloys, including high strength, toughness, corrosion resistance, and elevated-temperature performance, without significant size constraints. Therefore, investigating the correlation between the microstructure, physical phases, and wear resistance of multi-component alloys, and elucidating their wear mechanisms is crucial. Such research is theoretically significant for understanding material performance degradation and failure modes in marine environments, and practically important for the selection and optimization of friction components in offshore applications, thereby promoting the utilization of MPEAs as advanced metallic materials [14,15,16].

Multi-principal element alloys (MPEAs) demonstrate enhanced wear resistance; however, their performance, as quantified by the wear rate and coefficient of friction, is significantly influenced by alloy composition, processing methods, and testing parameters [17,18,19,20]. Wear rates for AlCrFe MPEAs have been reported within the range of 10^−4^ to 10^−7^ mm^3^·N^−1^·m^−1^, with friction coefficients varying from approximately 0.2 to 1. Liang et al. [17] synthesized “island-like” microstructures of Al_1.5_CrFeNiWTi_0.5_ on Q235 steel via laser cladding, resulting in a coating composed of body-centered cubic (BCC) and Laves phases. The Al_1.5_CrFeNiWTi_0.5_ coating notably improved the tribological properties of the Q235 steel substrate, exhibiting the lowest coefficient of friction (0.26) and wear rate (5.15 × 10^−7^ mm^3^·N^−1^·m^−1^) in deionized water. This enhancement is primarily attributed to the hydrolysis and oxidation reactions occurring on the coating surface, which increases the load-bearing capacity and prevents direct contact between the coating and the counterface, thereby reducing friction and wear. Research on MPEA wear resistance has largely focused on coatings, with some studies investigating the intrinsic properties of the alloys themselves. Joseph et al. [18] conducted pin-on-disk sliding wear experiments on CoCrFeMnNi and Al_x_CoCrFeNi at temperatures ranging from 25 °C to 900 °C, using Al_2_O_3_ balls and comparing the results with AISI 304 and Inconel 718. The wear mechanism transitioned from abrasive wear at room temperature to oxidation- and delamination-dominated wear above 600 °C. The wear behavior of AlCoCrFeNi was found to be similar to that of AISI 304. The wear resistance of AlCoCrFeNi significantly increased with temperature, surpassing that of Inconel 718 at 900 °C. This improvement in wear resistance above 600 °C was attributed to the formation of compact oxide scales in the contact zone and subsurface strengthening through fine-grained recrystallized structures containing precipitation-hardening phases [19]. Pouli et al. [20] investigated the wear resistance of MoCoCrFeNi and Mo_20_Ta_20_W_20_Nb_20_V_20_ alloys under various experimental conditions, demonstrating that the wear rate is highly dependent on these conditions.

This work investigates the Al_10_Cr_17_Fe_20_NiV_4_ alloy, focusing on the relationship between microstructural evolution and hardness/wear resistance. The study incorporates nanoalumina-reinforced phases to provide a comprehensive analysis, with the ultimate goal of establishing a theoretical and experimental foundation for novel, high-performance MPEAs.

## 2. Materials and Methods

The Al_10_Cr_17_Fe_20_NiV_4_ alloy, reinforced with nano-Al_2_O_3_, was fabricated via mechanical alloying followed by a plasma sintering process (SPS). The starting materials were high-purity aluminum, chromium, iron, nickel, and vanadium powders with a purity of over 99.5% and an initial particle size of 44 μm (200 mesh) (Al_2_O_3_, 100 nm, 99.99% purity, CMT New Material, Shanghai, China). Mechanical alloying was carried out in a SPEX 8000 vibratory mill (Houston, TX, USA) using hardened steel grinding media with a ball-to-powder weight ratio (BPR) of 10:1 and a ball milling time of 10 h. The subsequent SPS process had a heating rate of 100 °C min^−1^, an applied pressure of 25 MPa, and a sintering temperature of 1100 °C. Nano-alumina was added at 0, 1 wt.%, and 2 wt.% in Al_10_Cr_17_Fe_20_NiV_4_, which are abbreviated as MEA, MEA-1A, and MEA-2A, respectively.

The resulting microstructure was characterized using X-ray diffractometry (XRD), scanning electron microscopy (SEM), and transmission electron microscopy (TEM). A Zeiss Gemini SEM 500 (Jena, Germany), equipped with energy dispersive spectroscopy (EDS), and a JEOL 2100 TEM (Tokyo, Japan) were employed for detailed structural analysis.

Hardness measurements were conducted using a 5 N load and a 15 s holding time. Vickers hardness values were determined by averaging the results of 10 indentations performed on a polished surface. Friction and wear properties were assessed using an HT-1000 ball-on-disk wear tester (Lanzhou, China), applying a normal force of 10 N for a duration of 30 min. Each wear test was repeated at least three times to ensure the reproducibility of the results. The wear rates of disc and ball are calculated by

(1) $$W=\frac{∆m}{\rho \mathrm{FL}}$$

where Δ*m* is wear weight loss, kg; ρ is density, kg m^−3^; *F* is normal load, N; and *L* is total sliding distance, m. The density of samples was measured by Archimedes’ drainage method (ZMD-2, Shanghai, China).

## 3. Results

### 3.1. Transformation of Microstructures

**Figure 1:**
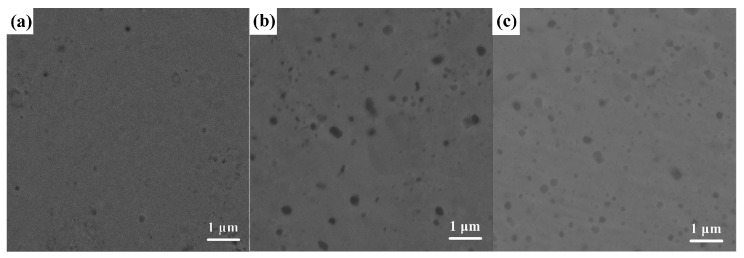
SEM for Al_10_Cr_17_Fe_20_NiV_4_-xAl_2_O_3_. (**a**) Al_10_Cr_17_Fe_20_NiV_4_, (**b**) Al_10_Cr_17_Fe_20_NiV_4_-1Al_2_O_3_, and (**c**) Al_10_Cr_17_Fe_20_NiV_4_-2Al_2_O_3_.

The density of Al_10_Cr_17_Fe_20_NiV_4_ alloy with nano-Al_2_O_3_ are 7.42, 7.39, 7.06 g·cm^−3^, respectively. To observe the microstructure of the Al_10_Cr_17_Fe_20_NiV_4_-xAl_2_O_3_ bulk alloy, SEM scanning was performed. Figure 1a–c show the SEM image of the Al_10_Cr_17_Fe_20_NiV_4_-xAl_2_O_3_ bulk alloy. It can be seen from the figure that the alloy has good densification during the sintering process, and there are no sintering defects, such as pores and gaps, on the alloy surface.

In Figure 1c, the aggregation phenomenon of dark areas on the surface of the Al_10_Cr_17_Fe_20_NiV_4_-2Al_2_O_3_ bulk alloy has been improved, indicating that the phase distribution in the alloy tends to be uniform.

**Figure 2:**
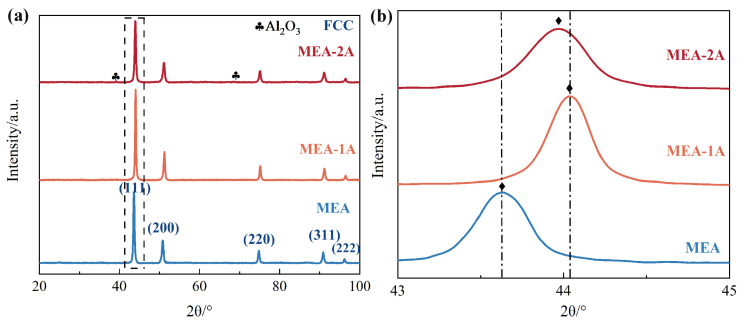
(**a**) XRD for Al_10_Cr_17_Fe_20_NiV_4_-xAl_2_O_3_ and (**b**) enlargement of the main peak (111).

The bulk densities of the Al_10_Cr_17_Fe_20_NiV_4_ alloy, following discharge plasma sintering, were determined to be 7.42 g/cm^3^, 7.39 g/cm^3^, and 7.06 g/cm^3^. Figure 2 presents the X-ray diffraction (XRD) spectra (a) and a magnified view of the primary peaks (b) for the Al_10_Cr_17_Fe_20_NiV_4_ alloy with varying nano-Al_2_O_3_ concentrations. The analysis of Figure 2a reveals that the Al_10_Cr_17_Fe_20_NiV_4_ alloy, along with Al_10_Cr_17_Fe_20_NiV_4_-1Al_2_O_3_, and Al_10_Cr_17_Fe_20_NiV_4_-2Al_2_O_3_ alloys, exhibits a face-centered cubic (FCC) structure. As depicted in Figure 2b, the primary peak positions of the alloys shift to higher angles with increasing nano-Al_2_O_3_ content, indicating a reduction in the lattice constant. However, the addition of 2 wt.% Al_2_O_3_ results in a leftward shift of the main peak and an increase in the lattice constant compared to Al_10_Cr_17_Fe_20_NiV_4_-1Al_2_O_3_. Despite the addition of Al_2_O_3_, the phase composition of the Al_10_Cr_17_Fe_20_NiV_4_-2Al_2_O_3_ bulk alloy remains largely unchanged compared to the Al_10_Cr_17_Fe_20_NiV_4_ alloy, although the Al_2_O_3_ content is significantly increased. Furthermore, the main peak position in Figure 2b is notably shifted to the left, with the lattice constant changing from 3.59 Å to 3.57 Å. These findings suggest that diffusion induces significant lattice distortion, primarily due to the atomic radii mismatch. Specifically, Al, with the largest atomic radius (1.43 Å, compared to Cr, Fe, Ni, and V at 1.27 Å, 1.27 Å, 1.24 Å, and 1.35 Å, respectively), contributes to severe lattice distortion upon its dissolution within the Al_10_Cr_17_Fe_20_NiV_4_ alloy’s lattice interstitials.

**Figure 3:**
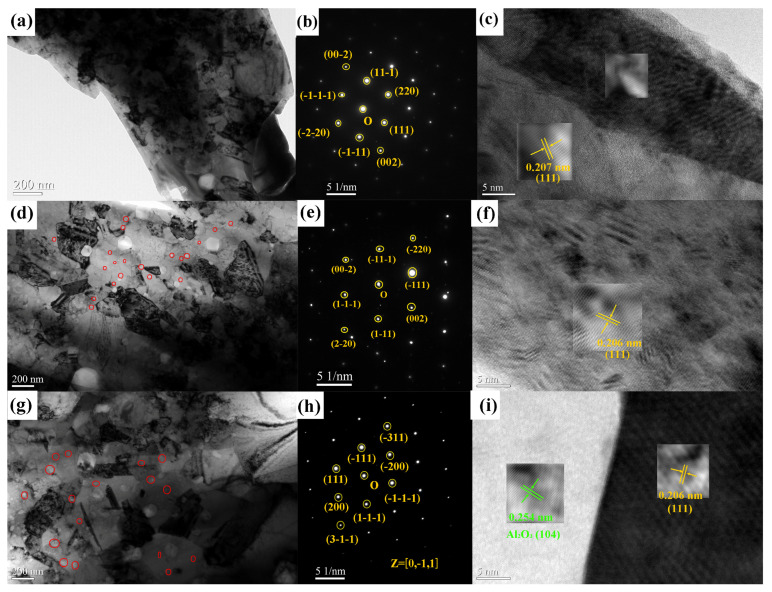
Al_10_Cr_17_Fe_20_NiV_4_ alloy TEM bright field image (**a**), Al_10_Cr_17_Fe_20_NiV_4_ alloy selected electron diffraction (SAED) map (**b**), Al_10_Cr_17_Fe_20_NiV_4_ alloy high-resolution TEM map (**c**); Al_10_Cr_17_Fe_20_NiV_4_-1Al_2_O_3_ alloy TEM bright field image (**d**), Al_10_Cr_17_Fe_20_NiV_4_-1Al_2_O_3_ alloy SAED map (**e**), Al_10_Cr_17_Fe_20_NiV_4_-1Al_2_O_3_ alloy high resolution TEM map (**f**), Al_10_Cr_17_Fe_20_NiV_4_-2Al_2_O_3_ alloy TEM bright field image (**g**), Al_10_Cr_17_Fe_20_NiV_4_-2Al_2_O_3_ alloy SAED map (**h**), and Al_10_Cr_17_Fe_20_NiV_4_-2Al_2_O_3_ alloy high resolution TEM map (**i**).

To elucidate the microstructural characteristics and phase composition of the Al_10_Cr_17_Fe_20_NiV_4_ matrix and its composites, the influence of nano- Al_2_O_3_ particles on the matrix structure was investigated via TEM. Figure 3a–c present the TEM bright-field image (a), selected area electron diffraction (b), and high-resolution image (c) of the Al_10_Cr_17_Fe_20_NiV_4_ alloy. These analyses reveal an FCC structure with a lattice constant of 0.357 nm, consistent with the XRD findings, indicating that the Al_10_Cr_17_Fe_20_NiV_4_ alloy is predominantly characterized by an FCC phase. The TEM images of the alloys with varying nano-Al_2_O_3_ particle contents are displayed in Figure 3d–i. Observations from Figure 3d–h indicate the presence of nanoparticles dispersed within the grains of the Al_10_Cr_17_Fe_20_NiV_4_-xAl_2_O_3_ alloy, with an estimated size of approximately 99 ± 8 nm for the nano-Al_2_O_3_ particles, which circled in red ring. A comparative analysis of the microstructures of Al_10_Cr_17_Fe_20_NiV_4_ and Al_10_Cr_17_Fe_20_NiV_4_-xAl_2_O_3_ reveals that both alloys primarily consist of face-centered cubic structures. The addition of nano-Al_2_O_3_ particles refines the grains of Al_10_Cr_17_Fe_20_NiV_4_-xAl_2_O_3_, thereby reducing the grain size. This grain size refinement contributes to grain boundary strengthening, which is the primary mechanism for enhancing hardness through the incorporation of nano-Al_2_O_3_ particles. The high-resolution images of the Al_10_Cr_17_Fe_20_NiV_4_ and Al_10_Cr_17_Fe_20_NiV_4_-xAl_2_O_3_ alloys demonstrate that the grain spacing aligns with the XRD results. The increased stress concentration at the grain boundaries is beneficial for improving the alloys’ toughness and wear resistance. At a specific sintering temperature, an increase in the superheat of the medium entropy alloy coupled with an increase in the cooling rate will inhibit the formation of nuclei, leading to the preferential growth of grains [21]. Furthermore, the residual stresses at the grain boundaries inhibit the growth of the FCC phase, resulting in a smaller grain size in Al_10_Cr_17_Fe_20_NiV_4_-xAl_2_O_3_ compared to Al_10_Cr_17_Fe_20_NiV_4_.

### 3.2. Mechanical Test

The microhardness values of the Al_10_Cr_17_Fe_20_NiV_4_ alloy matrix, as well as the composite material with varying weight percentages of nano-Al_2_O_3_ particles, are presented in Table 1. The base alloy exhibited a hardness of 727 HV. The incorporation of 1 wt.% nano- Al_2_O_3_ particles resulted in a significant increase in hardness, reaching 823 HV, which corresponds to a 13.2% enhancement. Conversely, the addition of 2 wt.% nano-Al_2_O_3_ particles yielded a hardness of 779 HV, representing a 6.7% increase compared to the matrix alloy.

**Figure 4:**
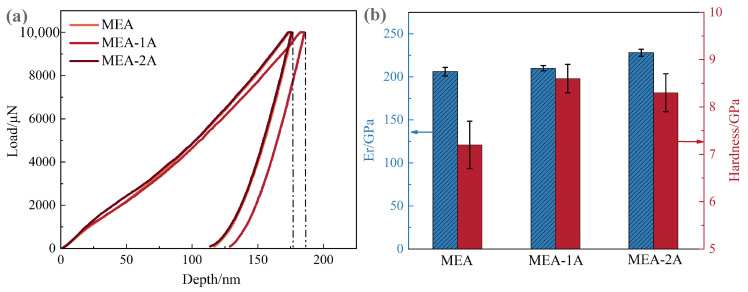
Nano-displacement and loading load profiles (**a**) and elastic modulus and hardness distributions (**b**) of Al_10_Cr_17_Fe_20_NiV_4_-xAl_2_O_3_ alloy with different nano-Al_2_O_3_ particle contents.

To investigate the mechanical characteristics of Al_10_Cr_17_Fe_20_NiV_4_-xAl_2_O_3_ alloys with varying nano-Al_2_O_3_ particle concentrations, nanoindentation experiments were performed. The load–displacement (L-D) curves, hardness, and Young’s modulus of the Al_10_Cr_17_Fe_20_NiV_4_-xAl_2_O_3_ alloys, under identical testing parameters, are presented in Figure 4. The curves distinctly exhibit three phases: an initial elastic loading phase, followed by elastic-plastic loading up to the maximum applied load, and finally, elastic unloading. A plateau is observed in the curves after the maximum load is reached, preceding the unloading phase. The elastic modulus values for the Al_10_Cr_17_Fe_20_NiV_4_-xAl_2_O_3_ alloy are 206 GPa, 210 GPa, and 228 GPa, respectively. Furthermore, the Al_10_Cr_17_Fe_20_NiV_4_-xAl_2_O_3_ alloy demonstrates high hardness, with values of 7.2 GPa, 8.6 GPa, and 8.3 GPa, respectively.

### 3.3. Wear Performance

**Figure 5:**
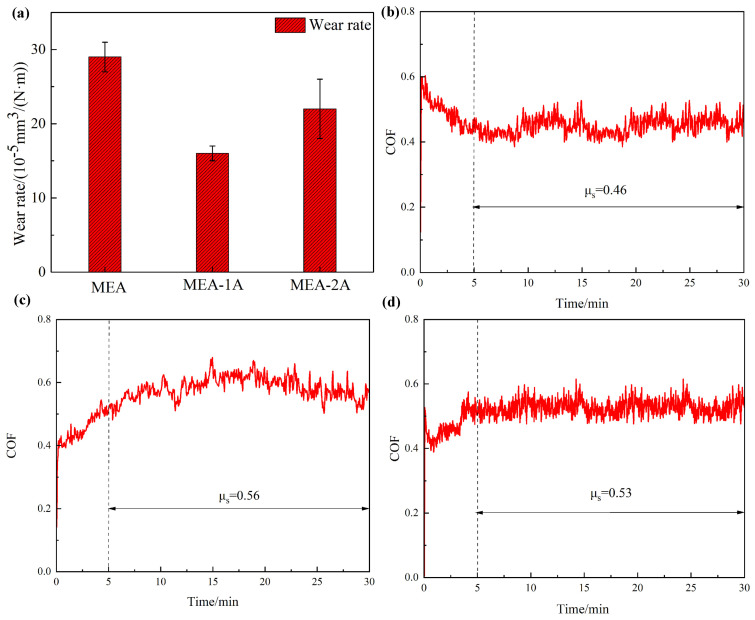
(**a**) Wear rate of Al_10_Cr_17_Fe_20_NiV_4_-xAl_2_O_3_ alloys; and (**b**–**d**) Friction coefficients during friction experiments’ schematic diagram of the alloys.

To assess the wear resistance of the Al_10_Cr_17_Fe_20_NiV_4_-xAl_2_O_3_ system alloys, friction and wear tests were performed on the alloy specimens. The wear rate and friction coefficient of the Al_10_Cr_17_Fe_20_NiV_4_-xAl_2_O_3_ system alloy during the friction experiment are presented in Figure 5. As shown in Figure 5a, the wear rate of the Al_10_Cr_17_Fe_20_NiV_4_ alloy is 29 ± 2 (10^−5^ mm^3^ N^−1^ m^−1^), whereas the wear rate of the Al_10_Cr_17_Fe_20_NiV_4_-1 Al_2_O_3_ alloy, with the addition of 1 wt.% nanoparticles, is 16 ± 1 (10^−5^ mm^3^ N^−1^ m^−1^), representing a 44.8% reduction. The wear rate for the Al_10_Cr_17_Fe_20_NiV_4_-2Al_2_O_3_ alloy was 22 ± 4 (10^−5^ mm^3^ N^−1^ m^−1^). Consequently, the wear rate of the alloy initially decreases and subsequently increases with the addition of nanoparticles. Figure 5b–d illustrate the real-time friction coefficients of the Al_10_Cr_17_Fe_20_NiV_4_-xAl_2_O_3_-based alloys. The evolution of the friction coefficient during wear friction typically comprises two phases: the break-in phase and the stable wear phase. The figure indicates that the alloys enter the stable wear stage after approximately 5 min of the grinding stage, where the friction coefficient stabilizes and fluctuations diminish. The average friction coefficients during the stable stage for the Al_10_Cr_17_Fe_20_NiV_4_-x Al_2_O_3_ system alloy are 0.46, 0.56, and 0.53, demonstrating that the wear rate is not directly correlated with the magnitude of the real-time friction coefficient.

## 4. Discussion

### 4.1. Effect of Nano-Al_2_O_3_ on Hardness

**Figure 6:**
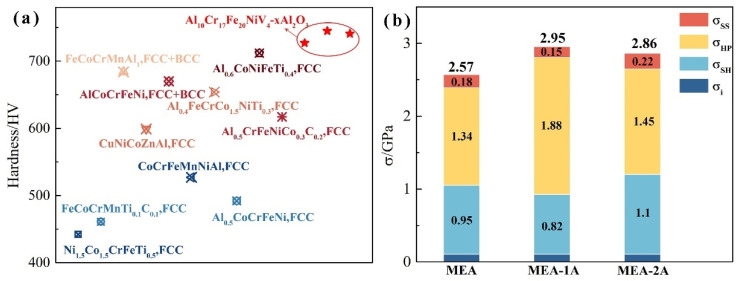
(**a**) Comparison of the hardness of Al_10_Cr_17_Fe_20_NiV_4_-xAl_2_O_3_ alloy with similar alloys and (**b**) the contribution of various influencing factors.

The mechanical properties of alloys can be enhanced by incorporating a minor volume fraction of a hard phase, a strategy applicable to the face-centered cubic (FCC) phase Al_10_Cr_17_Fe_20_NiV_4_ alloy. Hardness measurements on the Al_10_Cr_17_Fe_20_NiV_4_-xAl_2_O_3_ alloy yielded values of 727 HV, 823 HV, and 779 HV, respectively. These values, obtained from randomly selected areas, are considered representative of the alloy’s bulk mechanical behavior. Compared to the reported hardness of similar FCC-structured multi-principal element alloys [22,23,24,25,26,27,28], the Al_10_Cr_17_Fe_20_NiV_4_-xAl_2_O_3_ alloy exhibits superior mechanical properties, as illustrated in Figure 6.

To assess the influence of Al_2_O_3_ on the mechanical characteristics of the Al_10_Cr_17_Fe_20_NiV_4_ alloy, the Tabor equation was employed to estimate the stress contribution of each constituent. The Tabor equation, defined as *H* = 3*σ_flow_*, correlates the material’s hardness (*H*) with its flow stress (*σ_flow_*). The flow stress is attributed to the contributions of lattice friction stress on mobile dislocations, dislocation strengthening (Taylor strengthening), solid solution strengthening, and grain boundary strengthening (Hall–Petch strengthening). Consequently, the subsequent equations are utilized to quantify the various mechanisms contributing to the overall flow stress within the Al_10_Cr_17_Fe_20_NiV_4_-xAl_2_O_3_ alloy.

(2) $$\sigma_{flow}=\sigma_{i}+{∆\sigma }_{SH}+{∆\sigma }_{HP}+{∆\sigma }_{SS}$$

where *σ_i_* denotes the dislocation friction stress associated with lattice motion, approximately 105 MPa, while *σ_SH_* represents the cross-dislocation strengthening effect, reflecting the interaction between dislocations in coarser grains during deformation.

(3) $${∆\sigma }_{SH}={M\alpha Gb\rho }^{\frac{1}{2}}$$

where *M* is the Taylor’s index, and 3.06 is taken for both BCC structure and FCC structure; *α* is the material-specific correction coefficient, because the alloy material is a new material, and in this paper, α is assumed to be 1; *G* is the shear modulus, b is Bragg’s index, and ρ is the dislocation density, which is calculated from the XRD results. Therefore, the strain hardening *σ_SH_* due to high dislocation density in Al_10_Cr_17_Fe_20_NiV_4_-xAl_2_O_3_ alloy materials are 951, 823, and 1102 MPa, respectively.

*σ_HP_* is the strengthening due to grain size, also known as Hall–Petch strengthening, which can be calculated from Equation (4):

(4) $${∆\sigma }_{HP}=K_{HP}d^{-0.5}$$

where *K_HP_* is the Hall–Petch coefficient and d is the average grain size. Since the alloy is a new material and there is no literature to refer to for *K_HP_*, it was estimated by the law of mixtures using the *K_HP_* values of the constituent elements. *σ_HP_* and *K_HP_* were used to calculate the values of the constituent elements as 16, 100, 22, 454, and 203 MPa and 0.07, 0.60, 0.16, 0.95, and 0.21 MPa m^0.5^, respectively. Therefore, the *σ_HP_* of Al_10_Cr_17_Fe_20_NiV_4_-xAl_2_O_3_ alloy is 1344, 1882, and 1453 MPa.

The contribution of solid solution strengthening is represented by *σ_SS_*. In binary solid solution investigations, the elastic influence of solute atoms on dislocation activity and stress field distribution is a key factor in understanding the strengthening mechanism of materials. Established theoretical models of solid solution strengthening effectively elucidate this phenomenon and are applicable to all binary solid solution systems. Considering that medium-entropy alloys are complex multivariate alloys, Equation (5) was employed to estimate the solid solution strengthening of these complex systems.

(5) $${∆\sigma }_{SS}=AG\varepsilon^{\frac{4}{3}}C^{\frac{2}{3}}$$

where *A* is a dimensionless parameter taken as 0.1; *ε* is the lattice strain of the material, *G* is the shear modulus of the alloy, and *C* is the concentration of solute atoms taken as 0.16. The *σ_SS_* for the Al_10_Cr_17_Fe_20_NiV_4_-xAl_2_O_3_ alloy were 180, 153, and 224 MPa.

Based on the preceding estimations and Equation (2), the cumulative contribution of the various strengthening mechanisms to the flow stress (*σ_flow_*) of the Al_10_Cr_17_Fe_20_NiV_4_-xAl_2_O_3_ alloy is determined to be 2.57, 2.95, and 2.86 GPa, respectively. The corresponding hardness (H) values for the Al_10_Cr_17_Fe_20_NiV_4_-xAl_2_O_3_ alloy are 7.2, 8.6, and 8.3 GPa. In accordance with Tabor’s analysis, the H/*σ_flow_* ratio typically ranges from 2.4 to 3.2 for conventional materials. The calculated ratios for the Al_10_Cr_17_Fe_20_NiV_4_-xAl_2_O_3_ alloy are 2.80, 2.91, and 2.90, respectively, which are consistent with those observed in conventional polycrystalline materials. The contribution of the aforementioned strengthening mechanisms to the overall rheological stress of the Al_10_Cr_17_Fe_20_NiV_4_-xAl_2_O_3_ alloy is within an acceptable range. The results indicate that cross-dislocation-enhanced hardening and grain boundary strengthening are the predominant strengthening mechanisms in the Al_10_Cr_17_Fe_20_NiV_4_-xAl_2_O_3_ meso-entropic alloys, accounting for 89.1% of the rheological stress in the Al_10_Cr_17_Fe_20_NiV_4_ alloy. For the Al_10_Cr_17_Fe_20_NiV_4_-xAl_2_O_3_ alloy, the first two mechanisms contribute 91.5% and 89.2%, respectively. These two mechanisms exhibit a strong correlation with grain size; therefore, the incorporation of Al_2_O_3_ nanoparticles results in grain refinement, consequently influencing both cross-dislocation hardening and grain boundary hardening. Conversely, other mechanisms, such as solid solution strengthening, do not significantly contribute to the overall rheological stress of the Al_10_Cr_17_Fe_20_NiV_4_-xAl_2_O_3_ alloys.

### 4.2. Effect of Nano-Al_2_O_3_ on Wear Performance

**Figure 7:**
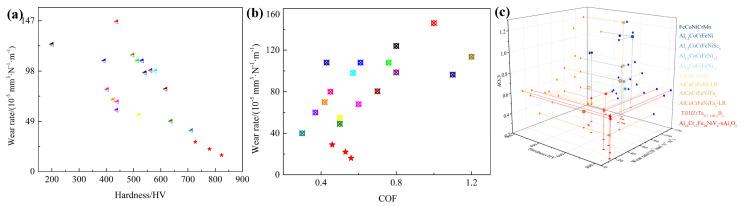
Hardness, coefficient of friction, and wear rate of alloy Al_10_Cr_17_Fe_20_NiV_4_-xAl_2_O_3_ compared to similar alloys: (**a**) H-Wear rate, (**b**) COF-Wear rate, (**c**) H-COF-Wear rate.

The friction coefficients for the Al_10_Cr_17_Fe_20_NiV_4_-xAl_2_O_3_ alloy were determined to be 0.57, 0.59, and 0.52, respectively, with corresponding wear rates of 29, 16, and 27 (10^−5^ mm^3^ N^−1^ m^−1^). A comparative analysis of the wear resistance of the Al_10_Cr_17_Fe_20_NiV_4_-xAl_2_O_3_ alloy was conducted against similar alloys, as illustrated in Figure 7 [28,29,30,31,32,33,34,35,36,37]. The data presented in these figures indicate that the Al_10_Cr_17_Fe_20_NiV_4_-xAl_2_O_3_ alloy exhibits superior hardness and wear resistance, suggesting favorable tribological properties.

**Figure 8:**
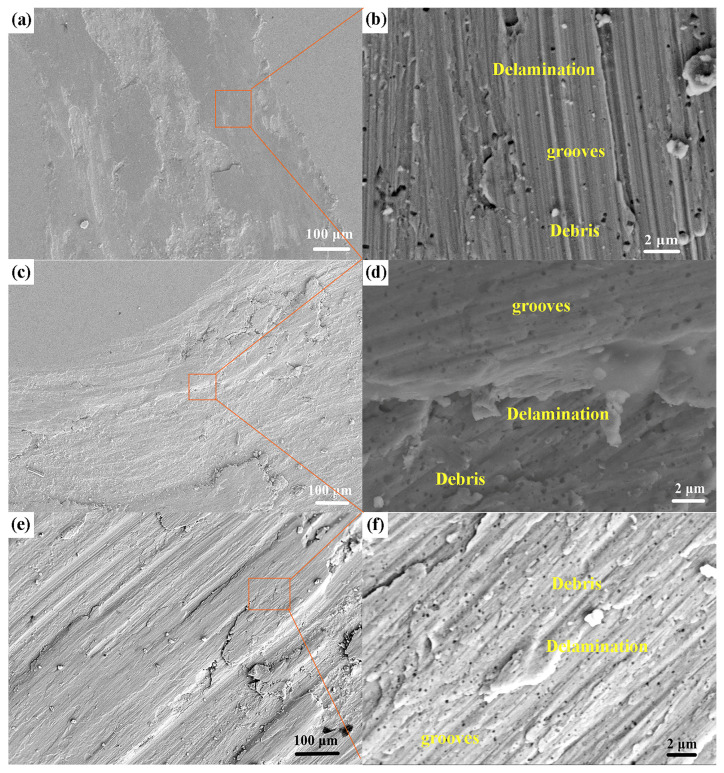
Different magnification worn surface SEM images of Al_10_Cr_17_Fe_20_NiV_4_-xAl_2_O_3_. (**a**,**b**) Al_10_Cr_17_Fe_20_NiV_4_, (**c**,**d**) Al_10_Cr_17_Fe_20_NiV_4_-_1_Al_2_O_3_, (**e**,**f**) Al_10_Cr_17_Fe_20_NiV_4_-2Al_2_O_3_.

To elucidate the influence of nano-Al_2_O_3_ particles on the frictional wear mechanism of the Al_10_Cr_17_Fe_20_NiV_4_-xAl_2_O_3_ alloy, a comprehensive analysis of the wear surface was conducted. SEM images of the wear surface of the Al_10_Cr_17_Fe_20_NiV_4_-xAl_2_O_3_ alloy are presented in Figure 8. At low magnification, the wear tracks appear shallow, with observable fragments and wear grooves. The incorporation of Al_2_O_3_ nanoparticles appears to enhance the matrix hardness, thereby providing wear resistance. Figure 8 reveals furrowing, debris, and delamination on the wear surface. The delamination is attributed to the hard Al_2_O_3_ particles inducing deformation and furrow formation on the relatively softer alloy surface. Furthermore, the presence of adhered wear debris suggests adhesive wear. Numerous fine grooves aligned with the sliding direction were also observed. The addition of nano-Al_2_O_3_ altered the surface characteristics, including a reduction in grooves and wear debris, alongside delamination and deformation. These observations suggest that the wear mechanism of the Al_10_Cr_17_Fe_20_NiV_4_-xAl_2_O_3_-based alloy primarily involves abrasive and delamination wear. The presence of delaminated areas and randomly distributed grooves, indicative of chip sliding and rolling, suggests two-body abrasive wear. The fine wear debris and increased oxygen content on the contact surface indicate the potential oxidation of the wear surface.

**Figure 9:**
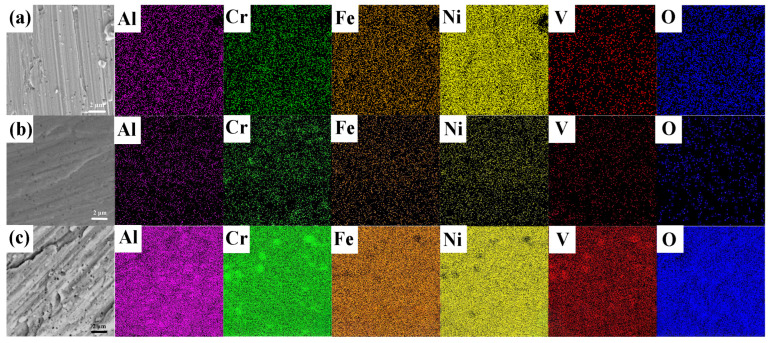
Worn surface SEM-EDS of Al_10_Cr_17_Fe_20_NiV_4_-xAl_2_O_3_. (**a**) Al_10_Cr_17_Fe_20_NiV_4_, (**b**) Al_10_Cr_17_Fe_20_NiV_4_-1Al_2_O_3_, (**c**) Al_10_Cr_17_Fe_20_NiV_4_-2Al_2_O_3_.

The SEM micrographs corresponding to the EDS images of the Al_10_Cr_17_Fe_20_NiV_4_-xAl_2_O_3_ system alloy wear surfaces are presented in Figure 9. The friction wear behavior, as evidenced by the EDS analysis, mirrors that of the Al_10_Cr_17_Fe_20_NiV_4_ multi-principal element alloy. The detection of oxygen in the EDS spectra suggests that the frictional heat generated during the wear process induces oxygen adsorption and subsequent oxidation reactions on the alloy surface.

Table 2 provides the quantitative elemental composition on the surface of each alloy sample. The EDS analysis reveals that the concentration of alloy matrix elements on the wear surface varies within a range of ±1.5 at.%, whereas the oxygen content exhibits a significant gradient. Specifically, the Al_10_Cr_17_Fe_20_NiV_4_ alloy displayed an oxygen content of 5.1 at.% on the wear surface. The introduction of 1 wt.% nano-Al_2_O_3_ particles (Al_10_Cr_17_Fe_20_NiV_4_-1Al_2_O_3_ alloy) resulted in a decrease in oxygen content to a minimum of 2.1 at.%. However, with a further increase in nanoparticles to 2 wt.% (Al_10_Cr_17_Fe_20_NiV_4_-2Al_2_O_3_ alloy), the oxygen content increased to 6.2 at.%. This non-monotonic trend aligns with the wear morphology characterization results: low oxygen content correlates with abrasive wear features, while a high oxygen content is associated with oxidative wear. The observed oxygen content characteristics can be attributed to the competitive mechanism between oxide film formation and abrasive oxidation during the friction process. The appropriate addition of nano-Al_2_O_3_ particles can effectively suppress surface oxidation caused by frictional heat through grain refinement and enhanced the matrix strength. The main component (1 wt% Al_2_O_3_) was retained as shown by the improvement in TEM and mechanical properties. The wear surface composition reflects dynamic wear processes (e.g., trioxidation reactions, particle removal, and matrix smearing), and localized Al and O signals were reduced despite the addition of Al_2_O_3_. The small difference in Al (0.2%) is within the uncertainty of the EDS, whereas the larger reduction in O is attributed to the dominant role of matrix-derived oxides, as well as the loss of Al_2_O_3_ particles during wear and the inaccuracy of lightweight elements in the EDS. Frictional contact generates localized temperatures exceeding 300–500 °C. These elevated temperatures promote the dissociation of oxygen molecules and chemisorption on active metal sites (Fe, Cr). The adsorbed oxygen then reacts with metal atoms, forming oxide nuclei (e.g., FeO, Cr_2_O_3_). Continued friction sustains heat input, promoting the thickening of the oxide layer until the mechanical removal of wear debris occurs. This cyclic process is consistent with the observed periodic fluctuations in the coefficient of friction and the coexistence of oxide patches and fresh metal regions in SEM images.

Layer-peeling wear, a process characterized by the initiation and propagation of subsurface cracks leading to material flaking under cyclic stress or friction, is dynamically modulated by the incorporation of nano-Al_2_O_3_ particles. The efficacy of this modulation is critically dependent on particle dispersion. Uniformly dispersed nano-Al_2_O_3_ particles act as impediments, directly hindering crack tip advancement. Upon crack propagation towards the particles, the stress field is dispersed, leading to energy absorption or crack deflection, thereby increasing the crack extension path and reducing the extension rate. A 44.8% reduction in wear rate at 1 wt.% suggests significant crack extension inhibition. Robust interfacial bonding between the particles and the matrix, achieved through metallurgical or chemical bonding, facilitates load transfer and mitigates preferential crack propagation along the interface. Furthermore, particle incorporation refines matrix grains, potentially through grain boundary pinning, thereby enhancing matrix toughness and inhibiting crack initiation. A 13.2% increase in hardness (823 HV) at 1 wt.% indicates enhanced resistance to deformation, consequently slowing fatigue damage accumulation. However, exceeding a critical particle concentration (e.g., 2 wt.%) leads to nano-Al_2_O_3_ agglomeration, forming micrometer-sized clusters. These clusters, characterized by weak interfacial bonding, act as stress concentration points. During friction, microcracks initiate around these clusters, rapidly evolving into macroscopic cracks. The wear rate at 2 wt.% increases to 22 × 10^−5^ mm^3^ N^−1^ m^−1^, approaching the matrix alloy’s level (29 × 10^−5^), indicating crack extension dominance in wear. The oxide film or transfer layer formed by excess particles on the wear surface exhibits a loose structure and high internal stress due to agglomeration. This brittle film is prone to localized spalling under frictional shear, generating abrasive debris (e.g., Al_2_O_3_ fragments), which exacerbates abrasive wear and promotes secondary cracking. Although the friction coefficient at 2 wt.% (0.53) is slightly lower than the substrate’s (0.57), the increased wear rate suggests that the film layer, despite reducing friction, fails to provide durable substrate protection due to its brittleness.

The correlation between hardness and wear resistance has been well documented in tribological research. A general principle holds that materials with higher hardness, such as ceramics and quenched steels, exhibit superior wear resistance due to enhanced resistance to plastic deformation. Classical studies confirm that materials with elevated Vickers hardness (HV) values demonstrate lower wear rates compared to softer counterparts. In cold-formed materials, refined relationships between HV and yield stress have been established through finite element simulations and experimental validation, achieving prediction errors below 10%. Similarly, studies on sintered steels reveal stable HV/tensile-strength ratios (~3.0–3.8), indicating predictable interconnections between mechanical properties.

However, hardness alone cannot universally predict wear behavior, as wear mechanisms (adhesive, abrasive, fatigue) are multifactorial processes influenced by material ductility, surface roughness, and environmental conditions. Consequently, Equation (6) was developed under specific experimental constraints (dry friction, room temperature) for a targeted material system (high-entropy alloys) and is not universally applicable. The model assumes isotropic hardening behavior and neglects environmental effects (oxidation, lubrication), potentially limiting its validity for materials with anisotropic microstructures or reactive environments.

**Figure 10:**
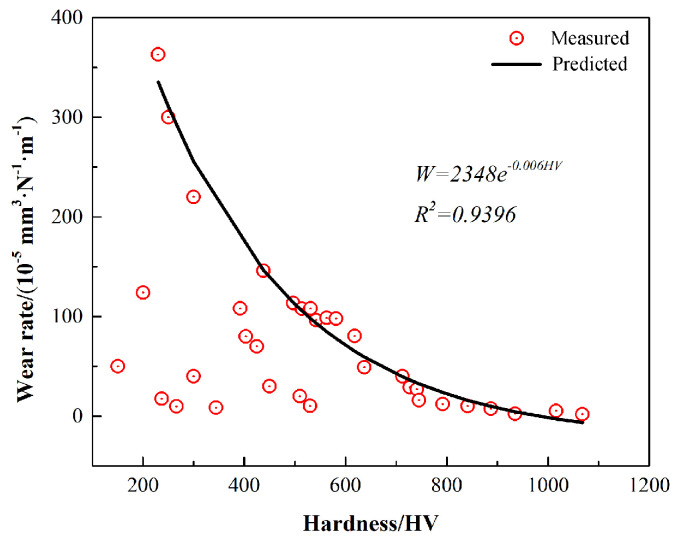
Fitting results of the alloy wear rate versus hardness formula.

Experimental validation involved calibrating the equation using wear data from three alloy variants (MEA, MEA-1A, MEA-2A) under identical testing conditions. Statistical analysis revealed a strong inverse correlation between the HV and wear rate within this dataset (R^2^ = 0.89), shown in Figure 10. Although Equation (6) is a phenomenological model, it aligns with energy dissipation theory: higher hardness reduces material removal rates by suppressing plastic deformation and subsurface crack initiation, consistent with the SEM observations of smoother wear tracks and finer debris particles in high-HV alloys. The equation is strictly applicable to nanocrystalline high-entropy alloys under dry sliding conditions, and its extension to other systems requires additional validation. Future comprehensive wear models must incorporate parameters such as fracture toughness and friction coefficients. Through extensive data fitting, the relationship between Vickers hardness and wear rate is quantitatively described by Equation (6):

(6) $$W=2348e^{-0.006\mathrm{HV}}$$

where *W* is the frictional wear rate of the alloy, 10^−5^ mm^3^ N^−1^ m^−1^, and HV is the Vickers hardness, HV.

Despite these limitations, Equation (6) retains significant practical value. It serves as an efficient screening tool during early-stage alloy development, enabling rapid performance ranking and guiding heat treatment/compositional adjustments to optimize hardness-wear resistance synergy. Such empirically derived correlations, when rigorously bounded, remain invaluable in applied research. The hardness–wear rate relationship defined by this equation provides a robust framework for expediting the evaluation of multi-principal element alloys’ wear resistance, thereby accelerating the development and application of novel materials.

## 5. Conclusions

The Al_10_Cr_17_Fe_20_NiV_4_-xAl_2_O_3_ alloy was successfully synthesized via mechanical alloying and subsequent SPS. The correlation between hardness and wear rate was investigated by incorporating the varying concentrations of alumina nanoparticles into the alloy matrix as a reinforcing phase. The results indicated that the Al_10_Cr_17_Fe_20_NiV_4_-xAl_2_O_3_ alloy exhibited a single-phase FCC structure, which was maintained with the addition of alumina nanoparticles. The hardness of the Al_10_Cr_17_Fe_20_NiV_4_ alloy without nano-alumina was 727 HV, with a corresponding wear rate of 29 (10^−5^ mm^3^ N^−1^ m^−1^). The incorporation of nano-alumina increased the hardness to 823 HV and significantly reduced the wear rate to 1.6 (10^−5^ mm^3^ N^−1^ m^−1^), representing a 45% reduction. The Al_2_O_3_ nanoparticles effectively mitigated alloy wear through crack passivation and matrix strengthening; however, excessive addition reversed this effect due to agglomeration-induced brittleness and thermal mismatch. The quantitative relationship between hardness (HV) and wear rate (W) was determined as W = 2348 e^−0.006HV^. Such carefully bounded empirical relationships, as demonstrated in the studies of cold-formed materials and dental enamel, remain valuable tools in applied research when accompanied by explicit scope limitations.

## Figures and Tables

**Table 1:** Room-temperature mechanical properties of Al_10_Cr_17_Fe_20_NiV_4_-xAl_2_O_3_ alloys.

Sample	Hardness/HV	*E_r_*/GPa	Hardness/GPa
MEA	727 ± 3	206 ± 5	7.2 ± 0.4
MEA-1A	823 ± 8	210 ± 3	8.6 ± 0.3
MEA-2A	779 ± 5	228 ± 4	8.3 ± 0.9

**Table 2:** EDS elemental content of Al_10_Cr_17_Fe_20_NiV_4_-xAl_2_O_3_ alloy wear surface (at.%).

Sample	Al	Cr	Fe	Ni	V	O
MEA	7.7	18.4	17.5	47.8	3.2	5.1
MEA-1A	7.5	18.5	19.1	48.9	3.9	2.1
MEA-2A	8.6	13.6	18.3	49.7	3.6	6.2

## Data Availability

Data are contained within the article.
